# A new species of *Cissampelos* (Menispermaceae) from Bolivia and Paraguay

**DOI:** 10.3897/phytokeys.38.6504

**Published:** 2014-06-04

**Authors:** Rosa del C. Ortiz, Michael H. Nee

**Affiliations:** 1 Missouri Botanical Garden, P.O. Box 299, St. Louis, MO 63166-0299, USA

**Keywords:** Bolivia, *Cissampelos*, conservation status, IUCN, Menispermaceae, Paraguay, sand dunes

## Abstract

The new species *Cissampelos arenicola* M. Nee & R. Ortiz, from the Bolivian and Paraguayan Chaco is described, its affinities are discussed, and its preliminary conservation status is evaluated. The species is at present known from 13 collections from sand dunes or dry forests. *Cissampelos arenicola* is distinguished from all other American species in the genus by its ovate- to subreniform-trilobed leaves, 8-locular synandria, and relatively large, and scarcely ornamented endocarps. The most common perianth condition in the pistillate flowers of *Cissampelos* is one sepal and one antesepalous petal, and while these may vary in number, they are always found adaxial to the carpel, and although the southern African taxon called *Cissampelos capensis*, whose generic position is uncertain, superficially resembles *Cissampelos arenicola*, its sepals and petals are consistently lateral to the carpel and not adaxial.

## Introduction

**Figure 1.**
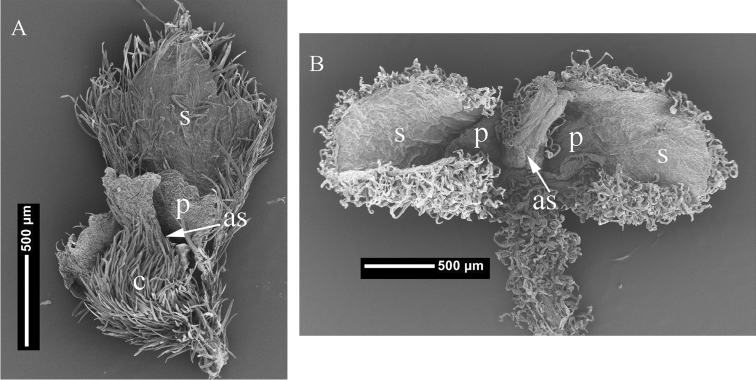
Pistillate flowers of **A** *Cissampelos arenicola* M. Nee & R. Ortiz, (*M. Nee 49044*, MO) **B** *Cissampelos capensis* L. f., (*S.L. Williams 295*, MO) showing locations of sepals and petals; s = sepal; p = petal; c = carpel; as = adaxial suture.

The pantropical genus *Cissampelos* L., together with African *Antizoma* Miers and the mostly Asian *Cyclea* Arn. ex Wight, were placed in subtribe Cissampelinae, one of the three subtribes in tribe Menispermeae, which together with seven other tribes were recognized by Diels (1910) in the family Menispermaceae. In studies based on *ndhF*, *matK*, *trnL-F*, *ITS*, *rbcL*, and *atpB* sequence data, subtribe Cissampelinae has been consistently recovered as monophyletic (Ortiz et al. 2007, Wang et al. 2007, Hoot et al. 2009, Jacques et al. 2011, Wang et al. 2012), although relationships within the subtribe remain unresolved (Jacques et al. 2011). The sampled species of *Cissampelos* form a clade that is sister to *Cyclea* (Ortiz et al. 2007, Wang et al. 2012, Ortiz et al. in prep.), but studies including the southern African *Cissampelos capensis* L. f. recovered a polyphyletic *Cissampelos*, with *Cyclea* (Hoot et al. 2009, Jacques et al. 2011), and *Antizoma* (Jacques et al. 2011) nested within. While *Cissampelos capensis* is recognized in *Cissampelos* (Botha 1980), at times it has been placed in *Antizoma* (Diels 1910), which is characterized by a shrubby habit, a spur borne along the stem [abaxial to the petiole], pistillate flowers with two sepals on opposite sides of the carpel, and two antesepalous petals (Miers 1851). The most common perianth condition in the pistillate flowers of *Cissampelos* is one sepal and one antesepalous petal, occasionally, more than one sepal and petal are found in some of the species (Diels 1910). Based on the fact that some species of *Cissampelos* display variation in sepal and petal number, the shrubby *Cissampelos capensis* was removed from *Antizoma* and included in the genus *Cissampelos* (Botha 1980). However, commonly overlooked is that while the pistillate flowers of *Cissampelos* may show variation in the number of sepals and petals, these are consistently located facing the adaxial slit of the carpel (Fig. 1A). On the other hand, the sepals and the antesepalous petals of *Antizoma* are usually located at both sides of the adaxial slit of the carpel (i.e., on the lateral sides of the carpel) (Fig. 1B), or around the carpel when more than two sepals/petals are present. The pistillate flowers of *Cissampelos capensis* have not only two sepals and two antesepalous petals but these are located in a similar manner as in *Antizoma angustifolia* Miers ex Harv. and in *Antizoma miersiana* Harv.

At present, the taxonomic status and phylogenetic affinities of *Cissampelos capensis* are unclear, similarly, the generic boundaries and relationships between *Cissampelos*, *Antizoma*, and *Cyclea* remain unresolved, and a re-assessment of all the genera in subtribe Cissampelinae is warranted, but is beyond the scope of this paper. Until further studies clarify the taxonomy and affinities of *Cissampelos capensis*, here it is still provisionally referred to as *Cissampelos* and we compare it to the new species *Cissampelos arenicola* as they both share superficial morphological resemblance in habit and foliage.

The last comprehensive revision of the genus *Cissampelos* recognized 20 species, nine of them distributed in the Americas, nine in Africa, one in Asia, and the pantropical *Cissampelos pareira* L. (Rhodes 1975). Species of *Cissampelos* are often found growing in open, disturbed habitats, and with a few exceptions, are morphologically variable, such that species limits are frequently difficult to discern. As new collections have accumulated since Rhodes’s (1975) revision, it has become evident that a number of collections from similar habitats in Bolivia and adjacent Paraguay, are conspecific but differ from all other described American species of Menispermaceae. Still, placing this material generically has been challenging. Because of the general similarity in leaf shape with the North American *Cocculus carolinus* (L.) DC., the specimens were initially identified as belonging to *Cocculus* DC. However, the Old World-centered *Cocculus* (Diels 1910, Troupin 1962), has only a few species in temperate and subtropical North America and Mexico, but none in South America. Examination of the minute staminate flowers of these Bolivian and Paraguayan plants allowed for the specimens to be confidently placed in *Cissampelos*, and the new species is described here as *Cissampelos arenicola*.

## Materials and methods

We studied all 13 herbarium collections of the new species housed at MO and at NY. Most of the collections have duplicates in several other herbaria but these were not available during this study. The specimens studied include male, female, and fruiting individuals, an infrequent situation in dioecious plants, such as Menispermaceae. Additionally, for comparison, we examined specimens for two other species that share some morphological similarities with the new species. These included: the Paleotropical *Cissampelos mucronata* A. Rich. (five specimens) and the southern African *Cissampelos capensis* (seven specimens). Measurements given in the species description refer to ranges of the mean values stemming from two to three replicate measurements of each structure and organ per individual voucher specimen. Before measuring, floral parts were first rehydrated, and endocarps were first boiled in water for a few minutes and the fleshy part of the fruit removed. Menispermaceae fruits develop asymmetrically so that the long axis do not necessary correspond with fruit length; endocarp measurement follow the convention of Ortiz (2012).

## Taxonomy

### Cissampelos arenicola

M. Nee & R. Ortiz sp. nov.

urn:lsid:ipni.org:names:77139692-1

http://species-id.net/wiki/Cissampelos_arenicola

Fig. 2

#### Diagnosis.

*Cissampelos arenicola* is distinguished from the remaining American species by its small, ovate- to subreniform-trilobed leaves and by its (6)8(10)-locular synandria. Its small leaves and viny habit superficially resemble the southern African *Cissampelos capensis* but differs by its (6)8(10)-locular synandria (vs. 4) and by sepals and petals located adaxial to the ventral slit of the carpel. The (6)8(10)-locular synandria of *Cissampelos arenicola* resembles that of African *Cissampelos mucronata*, from which it differs by its smaller (0.8–3 × 1.3–4 cm vs. 3.3–4.3 × 4.5–6.6 cm) leaves and its larger endocarp (6 × 7 mm vs. 4.3 × 4.7 mm).

#### Type.

**BOLIVIA.** Dpto. **Santa Cruz. Prov. Andrés Ibáñez:** along hwy from Santa Cruz to Abapó, 3 km S of crossing of railroad and 2 km S of bridge over Quebrada Peji, 17°58'00"S, 63°11'18"W, 450 m, 1 May 2001 (♂ fl), *M. Nee, S. Knapp & J. M. Mendoza 51717* (holotype USZ; isotypes LPB, MO-6393940, NY, and to be distributed to K).

**Figure 2.**
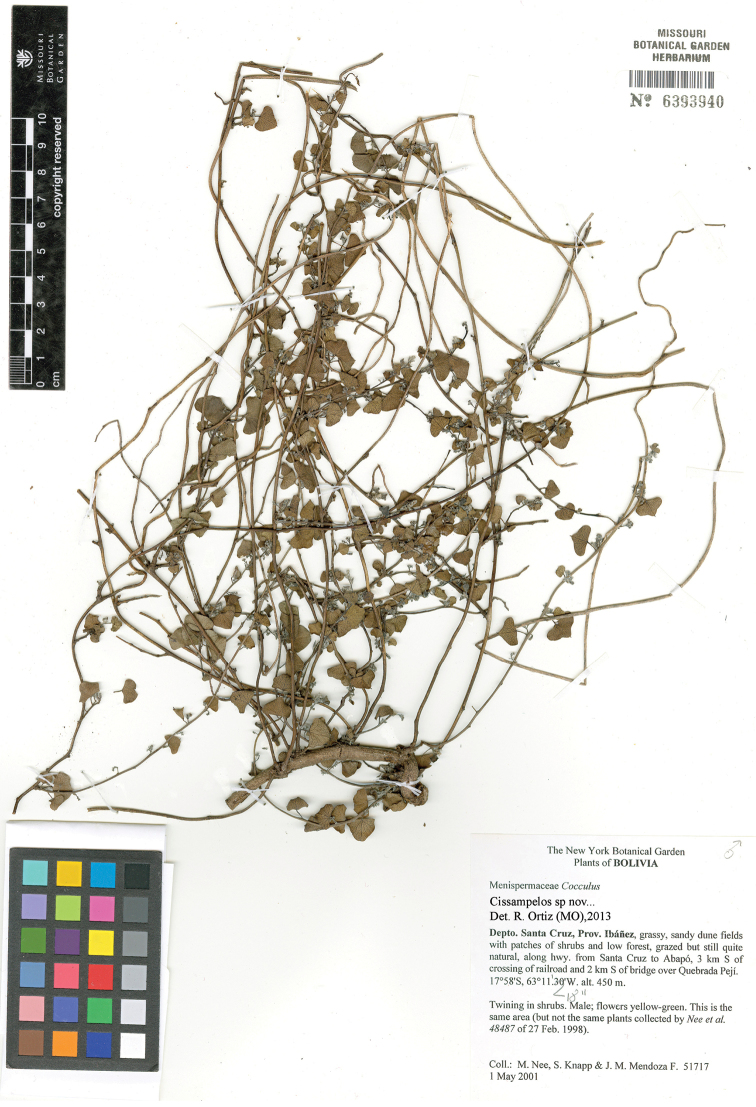
Isotype of *Cissampelos arenicola* M. Nee & R. Ortiz (*Nee et al. 51717*, MO).

#### Description.

**Figure 3.**
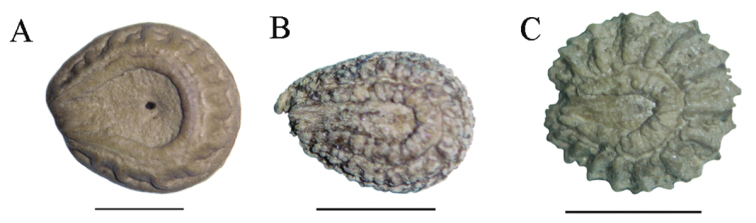
Endocarp ornamentation of **A** *Cissampelos arenicola* M. Nee & R. Ortiz (*F. Mereles & R. Degen 5075*, MO) **B** *Cissampelos capensis* L. f., (*W. Giess et al. 5247*, MO) **C** *Cissampelos mucronata* A. Rich., (*Muller & Biegel* 2281 MO). Scale bars = 3 mm.

*Twining*, perennial; stems striate, the older ones woody and glabrous, the younger ones subherbaceous and sparsely silvery-pilose, unarmed, growing in tangled viny masses to at least 5 m high in shrubs and small trees, to 5 mm in diameter; plants dioecious or infrequently monoecious. *Leaves* spiral, ovate- to subreniform-trilobed, usually broader than long, 0.8–3 × 1.3–4 cm, leaves associated with the inflorescences usually much smaller, lateral lobes divergent, rounded at apex, terminal lobe rounded and apiculate to aristate at the apex, chartaceous to subcoriaceous, glaucous, sparsely silvery-sericeous on both surfaces to nearly glabrous, palmately 6-nerved, basifixed or subpeltate with petiole inserted to 0.1–0.4 mm from the margin; petiole 5–17 mm long, pulvinulate at both ends. *Staminate* inflorescences: dichasium or monochasium, 1-2 from axils on adult or young leaves along the main stem or on young leaves of secondary axillary branches, silvery-pilose, peduncle 1.3–4.9 mm long, main axis of monochasium 1.2–3.2 mm long, bracts linear, 0.4–1.5 mm long; staminate flowers 5–6(–17); pedicels 0.5–1.6 mm long; sepals 4–6, 1.0–1.5 × 0.7–1.2 mm, obovate, shortly connate at base, concave, slightly spreading at anthesis, light cream-colored throughout, silvery-pilose abaxially, glabrous adaxially; petals usually 1, patelliform to barely cupuliform, 0.9–1.1 mm in diameter, or less frequently 3–4 and obovate, 0.7 × 1.1 mm, free, light cream-colored throughout, sparsely silvery-pilose abaxially, glabrous adaxially; synandrium 0.1–0.2 mm high, (6–)8(–10)-locular, loculi connate, transversely dehiscent, and radiating from a peltiform connective; carpellode absent. *Pistillate* inflorescences: 3–5 flowers fasciculate in the axils of adult or young leaves, sparsely silvery-pilose; pistillate flowers with pedicels 1.3–1.8 mm long; sepals and petals adaxial to the carpel; sepal 1(2), 1.2–1.5 × 0.9–1.2 mm, obovate, light cream-colored throughout, moderate silvery-pilose abaxially, less densely so adaxially; petals 1 (–3), subreniform, when 2 or 3, the petals free or partly to fully connate, opposite to the sepal, 0.7–0.9 × 0.9–1.4 mm, light cream-colored throughout, silvery-pilose abaxially, glabrous adaxially; sepal and petal soon deciduous, staminodes absent; carpel 1, gibbous, obliquely borne on pedicel, silvery-pilose, style 0.2–0.3 mm long, stigma 3–5-lobed, erect to spreading. *Drupe* globose, ca. 8 mm in diameter, glabrous, turning orange then red at maturity; mesocarp juicy; endocarp 6 × 7 mm, suborbicular-bilaterally compressed, with one tiny circular perforation on the lateral faces, ornamentation obscure, consisting of a very low medial ridge and obscurely transverse ridges (Fig. 3A); condyle bilaterally compressed, septiform (sensu Ortiz 2012).

#### Distribution and ecology.

**Figure 4.**
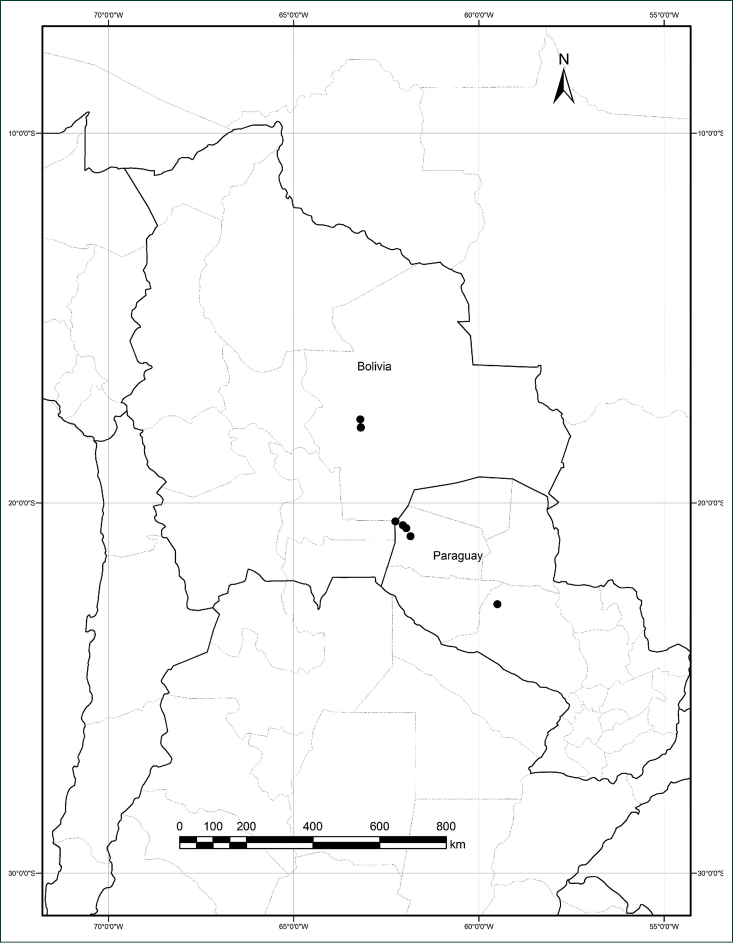
Distribution of *Cissampelos arenicola* M. Nee & R. Ortiz, based on examined collections with coordinates.

The species is at present known from southern Bolivia and northwestern Paraguay (Fig. 4). All Bolivian collections are from similar habitats of the sandy dune systems southwest of the city of Santa Cruz on the main highway which runs south to Abapó and on to Camiri and to Yacuiba on the Argentinian border. In Paraguay the species was collected along ruta Transchaco and also near the border with Bolivia in the proposed National Park Médanos del Chaco, where it has been found in seasonal forests and in dunes. In Bolivia there are extensive sandy savannas and large active dunes, the most well-known being the “Lomas de Arena” recreation area 15–20 km SSE of the center of Santa Cruz de la Sierra. The area where the species has been collected is on the western edge of this dune field. Plants were collected from 300–470 m elevation.

#### Pollination.

No observations of pollinators are available, nor notes on any possible odor. The extremely small size of the flowers suggests that a very small insect must be involved in pollination. Label note of *Nee 51401*: “It is difficult to separate the sparse and inconspicuous flowering and immature fruiting material from the vegetative mass”.

#### Phenology.

Male flowers were collected in February, May, November, and in December, pistillate flowers and mature fruits in February, April, June, and in December.

#### Etymology.

From the Latin, *arenicola*, dweller in sand, as the species seems to be restricted to sandy savanna soils and edges of dunes.

#### Preliminary conservation status.

*Cissampelos arenicola* is at present known from 13 collections at nine localities from southern Bolivia and northwestern Paraguay. The sandy savannas in the area surrounding Santa Cruz de la Sierra are frequently burnt and heavily grazed by cattle. This appears to have little effect on the native vegetation, but development of the land for subdivisions and chicken ranches is a greater threat and is destroying much of the original vegetation. Although the Viru-Viru pampa surrounding the international airport north of the city of Santa Cruz is maintained as a natural savanna, *Cissampelos arenicola* has never been collected there. In Paraguay, *Cissampelos arenicola* has been collected in habitats described as seasonal forest, but also in areas with nomadic sand dunes. Thus, the “ephemeral” condition described on the labels may refer to its dynamic and transient habitat and not to the plant per se. Because of the fragmented and threatened habitat in Bolivia, its reported ephemeral condition of its habitat in Paraguay, and its usually small population sizes, *Cissampelos arenicola* may be considered as vulnerable.

On the other hand, by applying the IUCN Red List Criteria (IUCN 2012), *Cissampelos arenicola* falls in the category of Endangered (EN) and meets the following criteria: A2c, as much as 50% decline of the population during the next 50 years inferred by the decline in habitat quality in the area of occupancy; B2c(ii,iii,iv), at present the area of occupancy is estimated as less than 500 km^2^, moreover, there are likely to be extreme fluctuations in the area of occupancy because of its unstable habitat.

#### Paratypes.

**Bolivia.** Santa Cruz: Prov. Andrés Ibáñez, along Quebrada Peji, vic. bridge of new hwy from Santa Cruz to Camiri and railroad bridge, 17°57'30"S, 63°11'00"W, 440 m, 11 Dec 1994 (imm & mat fr), *Nee 45861* (LPB, NY!, USZ); along hwy from Santa Cruz to Abapó, 3 km S of crossing of railroad and 2 km S of bridge over Quebrada Peji, 17°58'S, 63°11.3'W, 450 m, 27 Feb 1998 (♂ fl, imm fr), *Nee et al. 48487* (LPB, NY!, USZ); 15 Nov 2000 (♂ fl, imm fr), *Nee 51401* (NY); along hwy from Santa Cruz to Abapó, 5.4 km S of turnoff at “km 13”, 17°55'S, 63°15'W, 470 m, 18 Apr 1998 (imm fr), *Nee 49044* (LPB, NY!, USZ); Prov. Cordillera, a 3–4 km al S de Puerto Guaraní, al norte de la frontera Paraguaya, sabanas planas, partes húmedas, 20°30'S, 62°15'W, 400 m, 19 Jun 1992 (♀ fl & mat fr), *Mostacedo et al. 385* (MO!, USZ). **Paraguay.** Alto Paraguay: Fortín Teniente Montania, seasonal forests and swamps on clay soils, 22°03'15"S, 59°57'14"W, 5 Feb 2002 (♂ fl), *Zardini & Guerrero 57845* (MO!). Boquerón: Ruta Transchaco, a partir del km 702, entre PN Tte. Enciso y PN Médanos del Chaco, ambiente ruderal, 20°36'19"S, 62°02'54"W, 22 Feb 2006 (♂ fl), *De Egea et al. 923* (FCQ, BM, CTES, G, MO!, PY, SI, UNR); proposed National Park Médanos del Chaco, ephemerals on dunes, 20°41'03"S, 61°57'37"W, 300 m, 12 Dec 1998 (♂ fl), *Zardini & Duarte 49600* (AS, MO!); (mat fr), *Zardini & Duarte 49630* (AS, MO!); 13 Dec 1998 (♀ fl & imm fr), *Zardini & Duarte 49829* (AS, MO!). Nueva Asunción: 5 km después del Destacamento, sobre vegetación, 19 Mayo 1993 (♂fl), *Degen & Mereles 2969* (MO!). Presidente Hayes: Tyto Villazón, Fortín Guaraní, en espatillar arenoso, [22°44'S, 59°30'W], 2 Feb 1993 (♀ fl & mat fr), *Mereles & Degen 5075* (FCQ, MO!).

## Discussion

Although, the phylogenetic relationships of *Cissampelos* and the other genera in the Cissampelinae are still unresolved, *Cissampelos* is the oldest name in the clade and will therefore stand any future generic reassessment. While *Cissampelos arenicola* has not yet been included in any DNA based studies and as a result its phylogenetic affinities are not known, and despite the fact that the sepals and petals are variable in number, they are consistently located on the adaxial side of the carpel, a feature that is shared by all other studied species of *Cissampelos* with exception of *Cissampelos capensis*. Hence, we are confident that our new species belongs to *Cissampelos* s. str.

The genus *Cissampelos* is characterized by its dioecy, as are nearly all Menispermaceae. However, rare instances of monoecy have been reported by Miers (1871), and we observed monoecy in two collections (*Nee 48487*; *51401*) both from Bolivia, in which a few pistillate flowers and immature fruits were observed in the same inflorescence that is predominantly staminate. Petals of pistillate flowers in this inflorescence resemble these of staminate flowers in being cupuliform. There are reports of sex switching in Menispermaceae such as Asian *Tinospora cordifolia* (Willd.) Miers, where complete switching in a plant from staminate to hermaphrodite and back to staminate flowers have been observed (Geetha et al. 2007). Only plants with staminate flowers exhibit this labile sex expression, while plants with pistillate flowers show consistency in their expression (Geetha et al. 2007, Malpotra et al. 2009). The report of monoecy in the American species *Disciphania spadicea* Barneby is based on observations of a single collection from Jalisco (Mexico) (Carrillo-Reyes et al. 2013) which had both staminate and pistillate inflorescences on the same plant. Our case is similar to that of *Tinospora* where inflorescences with mostly staminate flowers may have a few pistillate flowers.

Anther cells in *Cissampelos arenicola* vary from 6–10 (Table 1), with about 45% of the sampled 24 staminate flowers having an 8-locular synandrium. This condition is also rarely observed in *Cissampelos grandifolia* Triana & Planch., but is unknown in the remaining American species of *Cissampelos* recognized by Rhodes (1975), which all have a 4-, rarely 6-locular synandrium. Similar 8-locular synandria are most commonly found in the vining *Cissampelos mucronata* A. Rich. from tropical Africa. *Cissampelos mucronata* differs from *Cissampelos arenicola*, however, in several characters, most obviously, larger leaves that are densely light golden hispidulous-tomentose on both surfaces. Vegetatively, *Cissampelos arenicola* superficially resembles the southern African *Cissampelos capensis*, from which it can be distinguished by the staminate flowers with a 6–10-locular (vs. 4-locular) synandrium, and pistillate flowers having sepals and petals located at the adaxial side of the carpel only. The larger and scarcely ornamented endocarp of *Cissampelos arenicola* (Fig. 3A) is readily distinguished from both, *Cissampelos capensis* (4.2 × 5.5 mm), and *Cissampelos mucronata* (Fig. 3B–C), (4.3 × 4.7 mm), respectively (Table 1).

**Table 1.** Main quantitative and qualitative variables that distinguish *Cissampelos arenicola* M. Nee & R. Ortiz from African species that are vegetatively similar and/or with 8-locular synandria.

Species	Habit	Leaf shape	Leaf size (cm)	Synandria locule #	Sepals & petals position regarding the carpel vs	Endocarp size (mm)
***Cissampelos arenicola***	vine	ovate-subreniform-trilobed	0.8–3 × 1.3–4	(6)8(10)	adaxially	6 × 7
***Cissampelos capensis***	shrub	ovate-triangular	1.1–3.9 × 0.8–2.8	4	laterally	4.2 × 5.5
***Cissampelos mucronata***	vine	ovate	3.3–4.3 × 4.5–6.6	(6)8(10)	adaxially	4.3 × 4.7

### Key to *Cissampelos arenicola* and African species vegetatively similar and/or with 8-locular synandria

**Table:** 

1	Inflorescences and flowers of both sexes densely to sparsely silvery-tomentose; staminate flowers with 4-locular synandria; pistillate flowers with sepals and petals located lateral to the adaxial slit of the carpel, often with staminodes	*Cissampelos capensis*
1’	Inflorescences and flowers of both sexes moderately silvery-pilose; staminate flowers with 6–10-locular synandria; pistillate flowers with sepals and petals located opposite to the adaxial slit of the carpel, lacking staminodes	2
2	Inflorescences of both sexes with well-developed, foliaceous bracts; perianth conspicuously brownish-speckled	*Cissampelos mucronata*
2’	Inflorescences of both sexes with small, ovate bracts; perianth pale cream-colored through	*Cissampelos arenicola*

### Key emphasizing endocarps of *Cissampelos arenicola* and African species vegetatively similar and/or with 8-locular synandria

**Table:** 

1	Endocarp 6 × 7 mm, lateral faces perforated	*Cissampelos arenicola*
1’	Endocarp 4.2 × 5.5 mm, lateral faces not perforated	2
2	Endocarp pyriform, surface ornamented with ridges and tubercles	*Cissampelos capensis*
2’	Endocarp subglobose, surface ornamented with well-developed transverse ridges	*Cissampelos mucronata*

## Supplementary Material

XML Treatment for Cissampelos arenicola
